# Bidirectional Electron-Transfer in Polypeptides with Various Secondary Structures

**DOI:** 10.1038/s41598-017-16678-7

**Published:** 2017-11-27

**Authors:** Ping Han, Ruiyou Guo, Yefei Wang, Lishan Yao, Chengbu Liu

**Affiliations:** 1 Department of Neurology, Haici Hospital Affiliated to Medical College of Qingdao University, Qingdao, 266033 Shandong P.R. China; 2 Qingdao Institute of Bioenergy and Bioprocess Technology, Chinese Academy of Sciences, Qingdao, 266101 Shandong P.R. China; 3 Institute of Theoretical Chemistry, School of Chemistry and Chemical Engineering, Shandong University, Jinan, 250100 Shandong China

## Abstract

The protein-mediated bidirectional electron transfer (ET) is the foundation of protein molecular wire, and plays an important role in the rapid detection of oxo-guanine-adenine DNA mismatches by MutY glycosylase. However, the influences of structural transitions on bidirectional ET are still not clear. In this work, the modified through-bond coupling (MTBC) model was further refined to correlate the structural transition and ET rate more quantitatively. With this model, various polyglycine structures (3_10_-helix, α-helix, β-sheets, linear, polyproline helical I and II) were studied to explore the influences of structural transitions on bidirectional ET. It was found that the HOMO-LUMO gaps (ΔE) in CN (from the carboxyl to amino terminus) direction are much lower than that in opposite direction, except for polypro I. However, with the equal tunneling energy, the differences between bidirectional ET rates are slight for all structures. In structural transitions, we found that the ET rates are not only affected by the Ramachandran angles, but also correlated to the alignment of C = O vectors, the alignment of peptide planes and the rearrangement of other structure factors. The detailed information can be used to rationalize the inhomogeneous ET across different protein structures and design more efficient protein molecular wires.

## Introduction

The assaults of endogenous and exogenous oxidative agents often lead to the oxidation of genomic DNA, which may cause aging, cancers, and neurological syndromes such as Alzheimer’s disease and amyotrophic lateral sclerosis^1–6^. A frequently observed oxidative damage is 7,8-dihydro-8-oxo-2′-deoxyguanosine (OG). It forms a stable base pair with 2′-deoxyadenosine (A), and would result in a G:C to T:A transversion mutation in replication^7^. Fortunately, the efficient repair systems for OG:A mismatch appear to be developed in organisms^8,9^. As the first and crucial step, MutY glycosylase specifically recognizes the mismatch and removes misincorporated adenine from DNA^10,11^.

As a human analog of the base excision repair (BER) enzymes, the adenine glycosylase activity and catalytic strategies of MutY have been investigated extensively^12–16^. Based on experimental studies, Barton *et al*.^15–18^ proposed an important model to elucidate the rapid detection and reorganization of MutY. If there is no DNA damage between two neighboring binding sites, the binding of one enzyme will drive electron-transfer (ET) to DNA duplex by the oxidation of inner [Fe_4_-S_4_]^2+^ cluster, and then DNA-mediated charge transfer (CT) will lead to reduction and redistribution of former bound MutY. In the presence of a mismatch, the DNA CT and the protein oxidation do not occur. The more strongly binding between the DNA duplex and the MutY in reduced state increases the likelihood of the enzyme approaching and repairing the lesion. In this model, the protein-mediated ET occurs in direction CN (from the carboxyl to amino terminus) or NC when MutY binds to or dissociates from DNA duplex. It indicates that the bidirectional ET in protein would play an important role in the recognizing and repairing process.

In addition, the protein molecular wire architectures were found in *Geobacter* and *Shewanella* bacteria recently^19,20^, and have gathered widespread interest^21–23^. It is also based on the ability of efficient bidirectional ET in protein. However, the influences of structural transitions on bidirectional ET are not clear, and the proteins are usually treated as homogeneous tunneling barriers.

In our previous works, a polypeptide model with S^−1^CH2CH2NH– head group was selected to study the bidirectional electron delocalizations and the influences of structural transitions on π*C = O energies, and a modified through-bond coupling (MTBC) model was proposed to correlate the structural detail and ET rate^24–26^. In this work, the MTBC model was further refined to reflect the influences of structural transitions on ET rate more quantitatively. With this model, we attempt to explore the ET differences through different areas of the same protein as well as ET difference along different directions. However, it is difficult to do high precision calculations for the large proteins. As is known to us, the biological functions of proteins mainly correlate with the special three-dimensional (3D) structures, and the 3D structures can be deconstructed into a limited number of secondary structural elements; i.e., helices, strands, and turns^27,28^. Accordingly, the study on polypeptide fragments with various secondary structures is a reasonable approximation to understand and mimic protein-related biological processes^29–31^. In addition, the bifunctional model proposed by Schlag *et al*.^32–34^ indicated that the ET in polypeptides should be controlled by the internal rotations of Ramachandran angles. This influence was further confirmed by a recent electrochemical study^35^. The investigation on different secondary structures should be helpful to explore the influences of structural transitions on ET.

Therefore, a series of model polypeptides with different secondary structures and changing lengths were constructed to investigate the protein-mediated bidirectional ET. In order to minimize the influence of donor and accepter on fragment structures and ET properties along different directions, the α-C radicals that are commonly found in peptides and proteins were adopted as donor and accepter^36–38^, as been used in the previous studies^39–41^. By analyzing the electronic structures and ET rates, the effects of structural transitions on bidirectional ET are discussed as well.

## Methods

### The refined MTBC model

**Figure 1:**
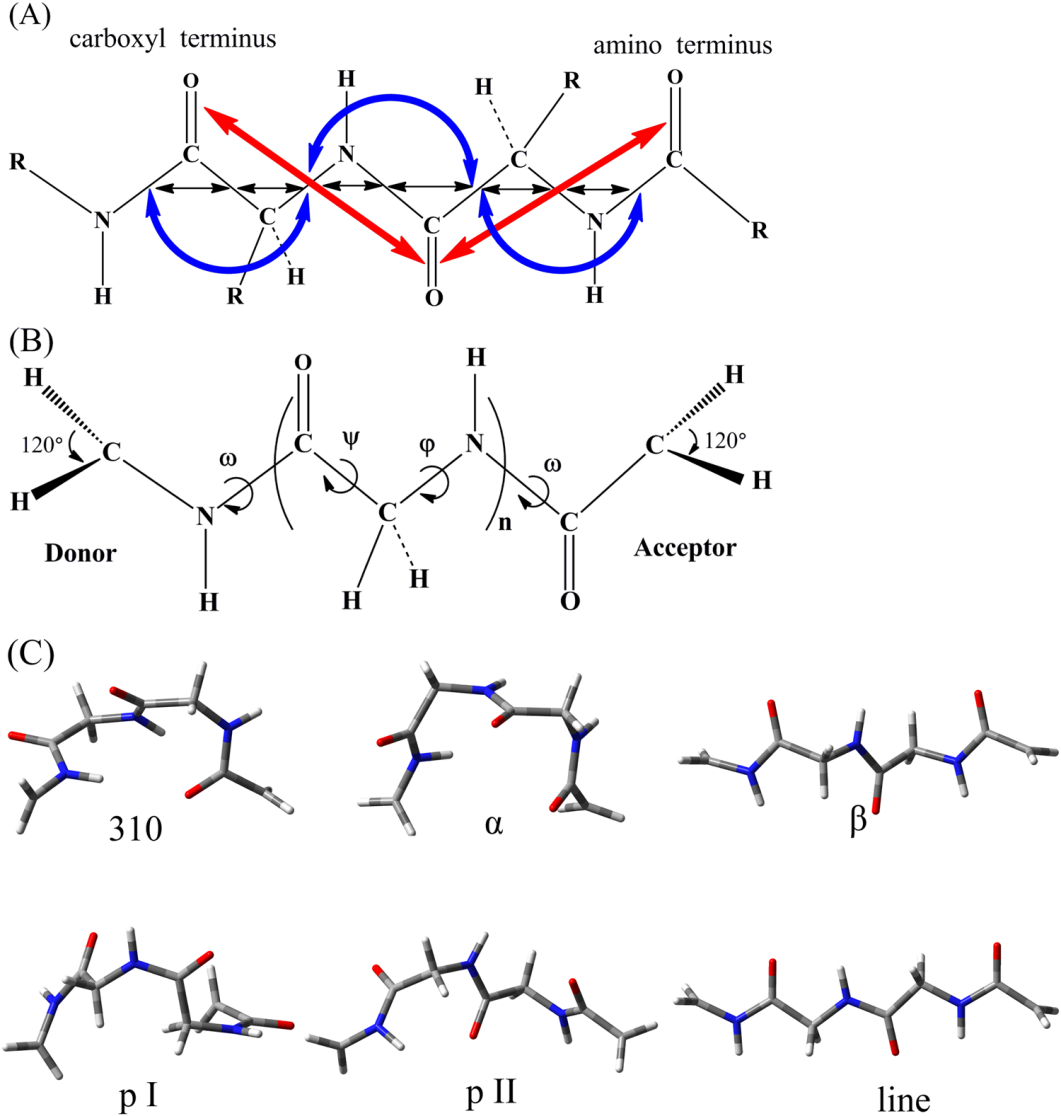
(**A**) The per-bond (black), per second neighbor bond (blue) and per C=O Pi (red) ET pathways of polypeptide chains. (**B**) The computational model with marked dihedrals that define the secondary structures. (**C**) The geometries of •CH_2_-NH(Gly)_2_CO-CH_2_• with 3_10_-helix (310), α-helix (α), β-sheets (β), polyproline helical I (p I) and II (p II), linear (line) secondary structures.

In proteins, the electronic interactions between donors and acceptors are usually rather weak, and the ET processes involve the electron couplings through peptide chains^42,43^. As discussed in our previous work^25^, the ET rates through different fragments of the same system should be correlated to their coupling strengths (∏ε) along tunneling pathway, and the decay factors (ε) obtained from MTBC model would be underestimated if minor contributions are neglected. In this work, all the couplings between bonding and antibonding orbitals as well as the couplings through per-bond, per second neighbor bond and per C = O Pi pathways (Fig. 1A) are treated as different contributions, and then are combined together as follows^39,44^.

1 $${\varepsilon }_{Bond,DihorPi}=-\frac{|{F}_{aa}|+|{F}_{ba}|}{E-{E}_{a}}+\frac{|{F}_{bb}|+|{F}_{ab}|}{E-{E}_{b}}$$

2 $${\varepsilon }_{T{\rm{otal}},Glyi}=({\varepsilon }_{Bond,1}\ast {\varepsilon }_{Bond,2}+{\varepsilon }_{Dih,1})\ast ({\varepsilon }_{Bond,3}+{\varepsilon }_{Dih,2}^{1/2})+{\varepsilon }_{Pi}$$

In equation (1), the *ε*_*Bond*,*Dih or Pi*_ represents the per-step decay factor along per-bond, per second neighbor bond or per C=O Pi pathway. *F*_*ij*_ is the Fock matrix element between bonding (b) and antibonding (a) orbitals from Natural Bond Orbitals (NBO)^44,45^ analysis. *E*, *E*_*a*_ or *E*_*b*_ represents the energy of tunneling electron, antibonding or bonding orbital respectively. As to equation (2), *ε*_*T*otal,*Glyi*_ is the total coupling strength through the *i* glycine unit. *ε*_*Bond*_, *ε*_*Dih*_ or *ε*_*Pi*_ represent the per-step decay factor along per-bond, per second neighbor bond or per C=O Pi pathway (Fig. 1A), and the coupling steps are marked with subscript 1, 2 or 3. The total coupling strength along a polypeptide chain can be given as

3 $${\varepsilon }_{T{\rm{otal}},{\rm{nGly}}}=\prod _{i=1}^{n}{\varepsilon }_{T{\rm{otal}},Glyi}$$

### Computational details

Six types of secondary structure (3_10_-helix, α-helix, β-sheets, linear, polyproline helical I and II) were chosen for the study of protein-mediated ET^27,46–48^. As shown in Fig. 1B, the model polypeptides were composed of α-C radicals and glycine units in the formula of •CH_2_-NH(Gly)_n_CO-CH_2_• (n=1~8). The ω, φ and ψ represent the dihedrals that define the specific secondary structures, and the corresponding values were given in Table 1. Taking •CH_2_-NH(Gly)_2_CO-CH_2_• as an example, the geometries of different secondary structures were shown in Fig. 1C.

**Table 1:** The dihedrals of particular secondary structures.

Structure	ω (°)	φ (°)	ψ (°)
3_10_-helix	180	−43	−24
α-helix	180	−67	−60
β-strand	180	−117	113
linear	180	180	180
polypro I	0	−95	160
polypro II	180	−64	126

All of the polypeptide structures were optimized using wB97X-D functional and 6-311+G(d) basis set^49,50^. In order to maintain the specific secondary structures, the ω, φ and ψ dihedrals were constrained during geometry optimizations^47,48^. Furthermore, the HCH bond angles of the neutral triplet biradical were fixed at 120°^39–41^. The NBO analyses were carried out at wB97X-D /6-311++G(d,p) level of theory, and the polarizable continuum model (PCM)^51^ with dielectric constant 4.0 was adopted to simulate the protein environment. The data for electronic structure analyses and the parameters for the refined MTBC model were extracted from NBO results directly. All the *ab initio* calculations were carried out using Gaussian09 software packages^52^.

## Results and Discussion

### Bidirectional ET rates in different secondary structures

As mentioned above, a main purpose of this work is to study the ET differences through different areas of the same protein. Since it is difficult to do high precision calculations for the large proteins, the typical secondary structures were treated as different fragments to study the ET differences approximately. The tunneling energy (*E*) is about −5 ~ −6 eV for typical biological donors and accepters, and can be tuned by changing the donor and acceptor structures^53–55^. In this work, the uniform tunneling energy −6 eV was adopted to evaluate the bidirectional coupling strengths of various polypeptide structures. In order to eliminate the influence of terminal groups, the relative decay factor through a glycine unit (*ε*_*ave*,*Gly*_) can be obtained as^40,41^

4 $${\varepsilon }_{ave,Gly}={(\frac{{\varepsilon }_{T{\rm{o}}{\rm{t}}{\rm{a}}{\rm{l}},({\rm{n}}+2){\rm{G}}{\rm{l}}{\rm{y}}}}{{\varepsilon }_{T{\rm{o}}{\rm{t}}{\rm{a}}{\rm{l}},{\rm{n}}{\rm{G}}{\rm{l}}{\rm{y}}}})}^{1/2}$$

where n and n+2 represent the number of glycine units in the polypeptide models. Then, the distance-dependent parameter (*β*) is calculated as^56^

5 $$\beta =-2ln{\varepsilon }_{ave,Gly}/{\rm{\Delta }}{r}_{ave,Gly}$$

where Δ*r*_*ave*,*Gly*_ is the average distance between carbonyl C atoms of neighboring units, defined as the effective ET distance.

As shown in Table 2, the calculated *β* values are about 1.32 and 1.06 Å^−1^ for α-helix and β-strand structures, in good agreement with the experimental data (1.3 and 1.1 Å^−1^)^56^. Furthermore, the calculated *β* parameters of other secondary structures (about 0.80, 1.06, 1.21 and 1.32 Å^−1^ for linear, polypro II, polypro I and 3_10_-helix) are also distributed within the range of experimental values (about from 0.80 to 1.4 Å^−1^)^57^. The results suggest that the MTBC model refined in this work is suitable to investigate the influences of structural transitions on protein-mediated ET.

**Table 2:** The distance-dependent parameter (*β*, Å^−1^) of different secondary structures.

n+2/ n		4/2	6/4	8/6	Average
3_10_-helix	CN	1.32	1.32	1.32	1.32
	NC	1.32	1.32	1.32	1.32
α-helix	CN	1.34	1.29	1.30	1.31
	NC	1.34	1.30	1.31	1.32
β-strand	CN	1.06	1.06	1.06	1.06
	NC	1.06	1.06	1.06	1.06
linear	CN	0.80	0.80	0.80	0.80
	NC	0.80	0.80	0.80	0.80
polypro I	CN	1.21	1.21	1.21	1.21
	NC	1.20	1.20	1.19	1.20
polypro II	CN	1.04	1.05	1.05	1.05
	NC	1.06	1.07	1.07	1.06

In addition, the average per-unit decay factors (*ε*_*ave*,*Gly*_) are reported in Table 3. The minor differences between bidirectional coupling strengths ascertains the shuttle function of protein molecular wire, and guarantees the rapid dissociation and redistribution of MutY in efficient DNA damage detection.

**Table 3:** The per-unit decay factors (*ε*_*ave*,*Gly*_, 10^−3^) of various secondary structures.

n+2/n		4/2	6/4	8/6	Average
3_10_-helix	CN	137.35	138.97	140.32	138.88
	NC	137.05	138.42	139.56	138.34
α-helix	CN	118.00	127.46	126.28	123.91
	NC	117.94	125.28	125.09	122.77
β-strand	CN	159.00	159.23	159.54	159.26
	NC	159.33	159.42	159.51	159.42
linear	CN	230.32	230.53	230.49	230.45
	NC	229.79	230.11	230.08	229.99
polypro I	CN	130.03	131.67	133.91	131.87
	NC	132.30	134.27	136.71	134.43
polypro II	CN	207.18	205.50	205.49	206.06
	NC	201.90	200.59	200.51	201.00

### The influences of structural transitions on protein-mediated ET

To explore the influences of structural transitions, the average per-bond decay factors were calculated for the three types of pathways (*ε*_*perBond*,*Bond*_, *ε*_*perBond*,*Dih*_, *ε*_*perBond*,*Pi*_). As reported in Table 4, the contributions of per-bond pathway (*ε*_*perBond*,*Bond*_) are similar (0.32 ± 0.02) in all structures, and the differences in coupling strength across different structures should be mainly resulted from the per second neighbor bond and per C=O Pi contributions.

**Table 4:** Decomposition of the per-bond decay factors (*ε*_*perBond*_).

n+2/ n		4/2				6/4				8/6			
		Bond	Dih	Pi	Tot	Bond	Dih	Pi	Tot	Bond	Dih	Pi	Tot
3_10_-helix	CN	0.31	0.38	0.27	0.52	0.31	0.38	0.27	0.52	0.31	0.39	0.27	0.52
	NC	0.31	0.38	0.27	0.52	0.31	0.38	0.27	0.52	0.31	0.38	0.27	0.52
α-helix	CN	0.30	0.29	0.35	0.49	0.31	0.29	0.38	0.50	0.31	0.29	0.37	0.50
	NC	0.31	0.29	0.35	0.49	0.31	0.29	0.37	0.50	0.31	0.29	0.36	0.50
β-strand	CN	0.33	0.41	0.26	0.54	0.33	0.41	0.26	0.54	0.33	0.41	0.27	0.54
	NC	0.33	0.41	0.26	0.54	0.33	0.41	0.26	0.54	0.33	0.41	0.27	0.54
linear	CN	0.34	0.49	0.28	0.61	0.34	0.49	0.28	0.61	0.34	0.49	0.28	0.61
	NC	0.33	0.49	0.28	0.61	0.33	0.49	0.28	0.61	0.33	0.49	0.28	0.61
polypro I	CN	0.32	0.36	0.28	0.51	0.32	0.36	0.28	0.51	0.32	0.36	0.29	0.51
	NC	0.32	0.36	0.28	0.51	0.32	0.36	0.29	0.51	0.32	0.36	0.30	0.51
polypro II	CN	0.32	0.39	0.43	0.59	0.32	0.39	0.43	0.59	0.32	0.39	0.43	0.59
	NC	0.32	0.39	0.43	0.59	0.32	0.39	0.42	0.59	0.32	0.39	0.42	0.59

**Figure 2:**
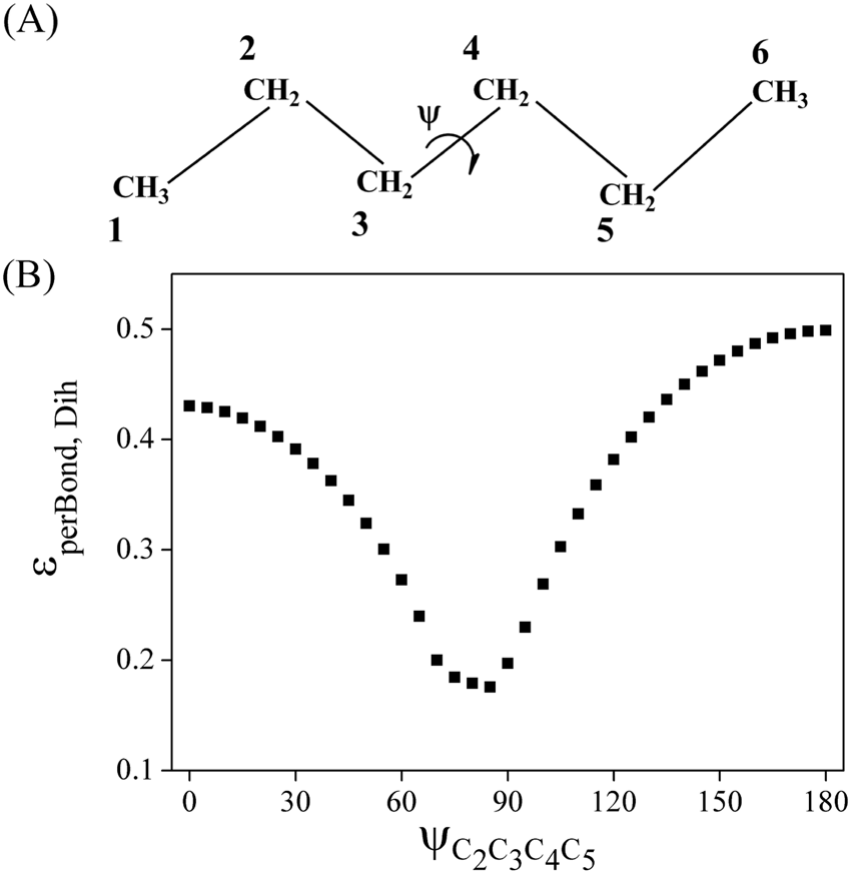
(**A**) The n-hexane model and (**B**) the effect of ψ_C2C3C4C5_ rotation on C_2_-C_3_/C_4_-C_5_ coupling.

As to the per second neighbor bond pathway, the coupling strength is correlated to the rotations of dihedral angles. Take n-hexane as an ideal example (Fig. 2A), the effect of rotation around C_3_-C_4_ bond on C_2_-C_3_/C_4_-C_5_ coupling was investigated by constrained optimizations and the refined MTBC model. As shown in Fig. 2B, the *ε*_*perBond*,*Dih*_ values of E at −6 eV decrease when ψ_C2C3C4C5_ rotates from 0° to 85° (at an interval of 5°), and then increase a little faster when ψ_C2C3C4C5_ rotates from 85° to 180°. Accordingly, the *ε*_*perBond*,*Dih*_ values of various polypeptide structures would increase in the order of α-helix < polypro I < 3_10_-helix,polypro II < β-strand < linear (Table 4). In addition, it was found that the energies of antibonding and bonding orbitals change less than ±2‰ in rotation, and the difference in coupling strength should be mainly attributed to the change of |*F*_*ij*_|.

**Figure 3:**
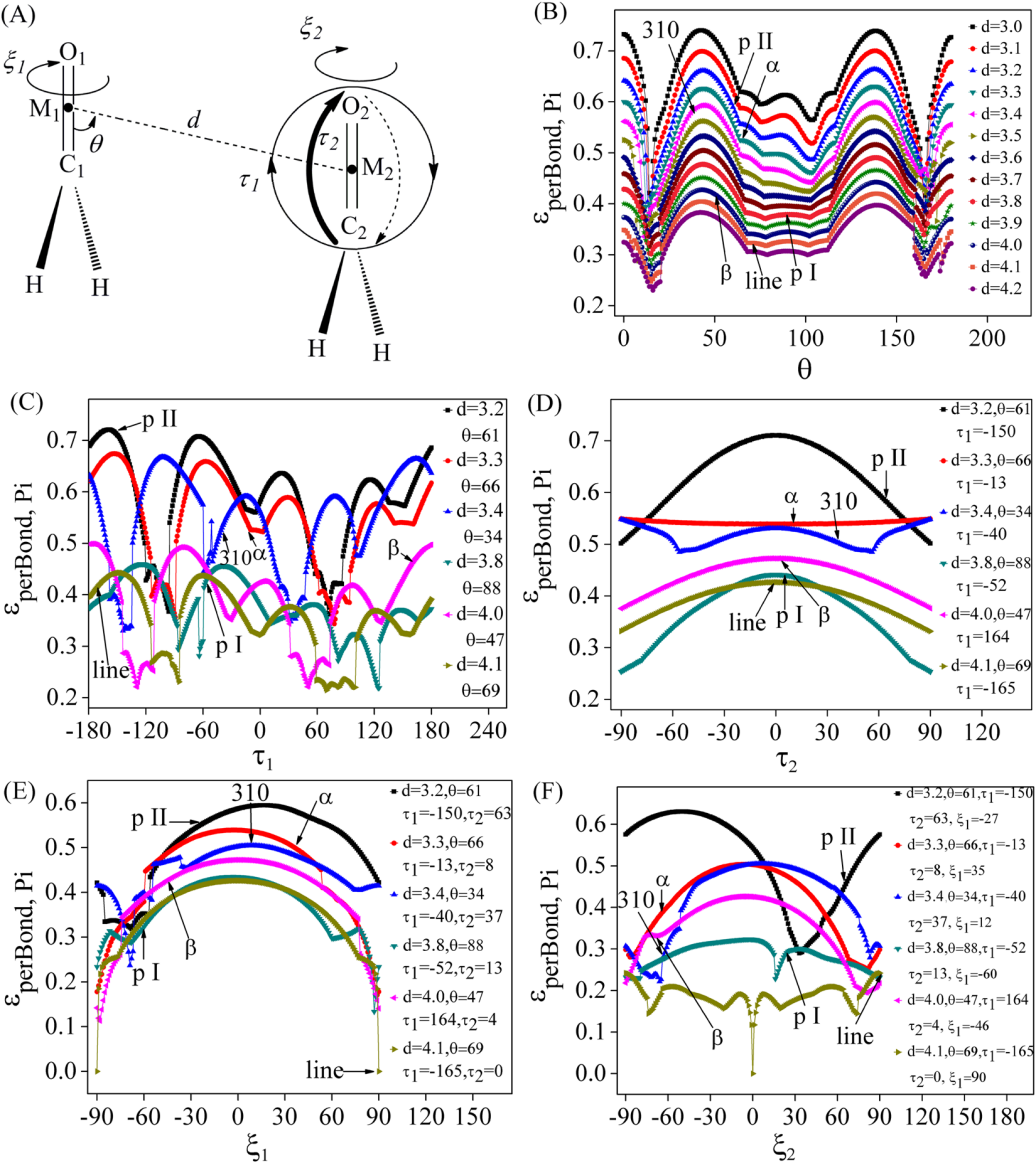
(**A**) The simplified formaldehyde model with 6 parameters (*d*, *θ*, *τ1*, *τ2*, *ζ1* and *ζ2*) that are used to define the alignment of C=O vectors and peptide planes approximatively. Then, (**B**) *d* and *θ*, (**C**) *τ1*, (**D**) *τ2*, (**E**) *ζ1* and (**F**) *ζ2* were scanned orderly to ascertain their influences on decay factors in structural transitions. The sites of 310-helix (3.4 Å, 34°, −40°, 37°, 12°, −64°), α-helix (3.3 Å, 66°, −13°, 8°, 35°, −67°), β-strand (4.0 Å, 47°, 164°, 4°, −46°, −68°), linear (4.1 Å, 69°, −165°, 0°, 90°, 90°), polypro I (3.8 Å, 88°, −52°, 13°, −60°, 24°) and polypro II(3.2 Å, 61°, −150°, 63°, −27°, 69°) structures were labeled.

As to per C=O Pi pathway, we found that the coupling strength is not only correlated to the alignment of C=O vectors, but also affected by the alignment of peptide planes and the rearrangement of polypeptide structures. Taking two optimized formaldehyde molecules as an ideal model, 6 parameters (*d*, *θ*, *τ1*, *τ2*, *ζ1* and *ζ2*, Fig. 3A) were used to describe the alignment of C=O vectors and peptide planes approximatively, where *d* represents the distance between the midpoints (M1 and M2) of neighboring C=O bonds, *θ* is the angle between C1=O1 and M1-M2, *τ1* indicates the C2=O2 rotation around M2 inner C1-O1-M2 plane, *τ2* represents the C2=O2 rotation inner vertical plane, *ζ1* indicates the rotation of the first fragment around C1-O1 axis, and *ζ2* represents the rotation of the other fragment around C2-O2 axis. The *d*, *θ*, *τ1* and *τ2* parameters reflect the alignment of C=O vectors, while *ζ1* and *ζ2* represent the alignment of peptide planes. According to the alignments along CN direction, the average parameters between n and n+1 peptide planes are about (3.4 Å, 34°, −40°, 37°, 12°, −64°), (3.3 Å, 66°, −13°, 8°, 35°, −67°), (4.0 Å, 47°, 164°, 4°, −46°, −68°), (4.1 Å, 69°, −165°, 0°, 90°, 90°), (3.8 Å, 88°, −52°, 13°, −60°, 24°) and (3.2 Å, 61°, −150°, 63°, −27°, 69°) for 310-helix, α-helix, β-strand, linear, polypro I and polypro II structures respectively. Therefore, the *d* (from 3.0 to 4.2 Å with an interval of 0.1 Å), *θ* (from 0 to 180° with an interval of 1°), *τ1* (from −180 to 180° with an interval of 1°), *τ2* (from −90 to 90° with an interval of 1°), *ζ1* (from −90 to 90° with an interval of 1°) and *ζ2* (from −90 to 90° with an interval of 1°) were scanned orderly to ascertain their influences on decay factors (E = −6 eV) in structural transitions.

Taking *ε*_*perBond*,*Pi*_ along CN direction as an example, the changing curves were shown in Fig. 3B to Fig. 3F, and the corresponding sites of different secondary structures were marked. It was found that the *ε*_*perBond*,*Pi*_ values decrease with the increasing distance *d* (Fig. 3B). As to *θ*, the highest sites appear around 0°, 45°, 135° and 180°, while the lowest sites appear around 15° and 165° (Fig. 3B). For *τ1*, the curve shapes are similar, but the four peaks shift obviously for different structures. The highest sites appear around (−105°/165°), (−155°/−55°), (−175°/−80°), (−145°/−60°), (−125°/−40°) and (−160°/−65°) for 310-helix, α-helix, β-strand, linear, polypro I and II structures respectively (Fig. 3C). As to *τ2* (Fig. 3D), the *ε*_*perBond*,*Pi*_ values decrease with the deviation of C2=O2 from C1-O1-M2 plane for β-strand (*τ1* = 164°), linear (*τ1* = −165°), polypro I (*τ1* = −52°) and polypro II (*τ1* = −150°). For 310-helix (*τ1* = −40°), α-helix (*τ1* = −13°), the decay factors decrease first and then increase with the deviation of C2=O2 from C1-O1-M2 plane. As to *ζ1* and *ζ2*, since the initial structures vary considerably, the curve shapes are different. Taking α-helix (*τ1* = −13°, *τ2* = 8°) and β-strand (*τ1* = 164°, *τ2* = 4°) model as an example, in which the two H-C-O planes are almost parallel packing, the decay factors would decrease with the deviation from parallel packing (Fig. 3E,F). In addition, we noticed that there are some sharp discontinuities on the *τ1* scanning curves. Taking 310-helix (blue line in Fig. 3C) and β-strand (pink line in Fig. 3C) model as an example, the Fock matrix elements and the orbital energies for the discontinuity points (−159/−158, −135/−134, −60/−59, 47/48 for 310-helix and −145/−144, −112/−111, 31/32, 73/74 for β-strand) were reported in Table 5. It was found that the energies of antibonding and bonding orbitals change slightly (less than ±7%), and the significant difference in coupling strength (about ±13% ~ ±54%) should be mainly attributed to the change of |*F*_*ij*_|.

**Table 5:** The Fock matrix elements and the orbital energies (eV) for the discontinuity points in *τ1* scan of 3_10_-helix and β-strand model (Fig. 3C).

	*τ1*	*|F*_*b1b2*_ *|*	*|F*_*b1a2*_ *|*	*|F*_*a1a2*_ *|*	*|F*_*a1b2*_ *|*	*E*_*b1*_	*E*_*a1*_	*E*_*b2*_	*E*_*a2*_
3_10_-helix	−159	0.0003	0.0178	0.0388	0.0068	−150.16	29.53	−228.96	99.92
	−158	0.0019	0.0126	0.0140	0.0073	−149.60	29.06	−232.62	100.61
	−135	0.0048	0.0020	0.0083	0.0083	−138.69	19.13	−227.64	99.72
	−134	0.0036	0.0243	0.0401	0.0085	−138.70	19.11	−232.69	104.43
	−60	0.0060	0.0028	0.0193	0.0264	−229.64	102.00	−138.27	17.67
	−59	0.0061	0.0013	0.0111	0.0069	−231.71	103.92	−138.18	17.54
	47	0.0078	0.0032	0.0109	0.0150	−130.85	25.88	−230.03	99.96
	48	0.0013	0.0144	0.0301	0.0068	−130.90	25.87	−232.16	102.57
β-strand	−145	0.0012	0.0086	0.0196	0.0038	−143.00	22.40	−224.83	96.09
	−144	0.0004	0.0047	0.0069	0.0023	−143.01	22.39	−233.08	103.21
	−112	0.0016	0.0000	0.0052	0.0027	−141.01	20.57	−228.66	99.32
	−111	0.0020	0.0097	0.0198	0.0039	−141.03	20.61	−232.17	102.56
	31	0.0008	0.0046	0.0134	0.0053	−136.46	24.77	−227.50	99.45
	32	0.0014	0.0026	0.0062	0.0033	−136.47	24.82	−231.69	103.41
	73	0.0024	0.0003	0.0042	0.0040	−133.70	22.32	−225.78	98.09
	74	0.0017	0.0050	0.0130	0.0058	−133.72	22.29	−233.44	104.59

Furthermore, it was found that the *ε*_*perBond*,*Pi*_ values of formaldehyde models truncated from polypeptide chains are different from the values of whole chain models (Table 6). Thus, the rearrangement of other structure factors maybe also affect the *ε*_*perBond*,*Pi*_ values, and the ratio (polypeptide model/ truncated formaldehyde model) is 0.79, 0.96, 0.79, 1.14, 1.09 or 0.99 for 310-helix, α-helix, β-strand, linear, polypro I or polypro II structure respectively. Integrating all factors, the *ε*_*perBond*,*Pi*_ values decrease in the order of polypro II> α-helix> polypro I, linear, 3_10_-helix, β-strand.

**Table 6:** The *ε*_*perBond*,*Pi*_ values of formaldehyde models truncated from polypeptide chains (Truncated) as well as the average values from the whole chains (Whole).

		Truncated	Whole
3_10_-helix	CN	0.34	0.27
α-helix	CN	0.39	0.37
β-strand	CN	0.34	0.27
linear	CN	0.24	0.28
polypro I	CN	0.27	0.29
polypro II	CN	0.43	0.43

In addition, it is necessary to point out that the refined MTBC model is based on the hypothesis that the polypeptide structures do not change in ET process. For ET systems with obvious structure fluctuation, the model should be used with enough sampling structures, and the accuracy remains to be tested. As reported above, the data from MTBC model are sensitive to many structural parameters, the results here from average structures may be changed in these systems.

### Bidirectional HOMO-LUMO gaps in various polypeptide structures

**Figure 4:**
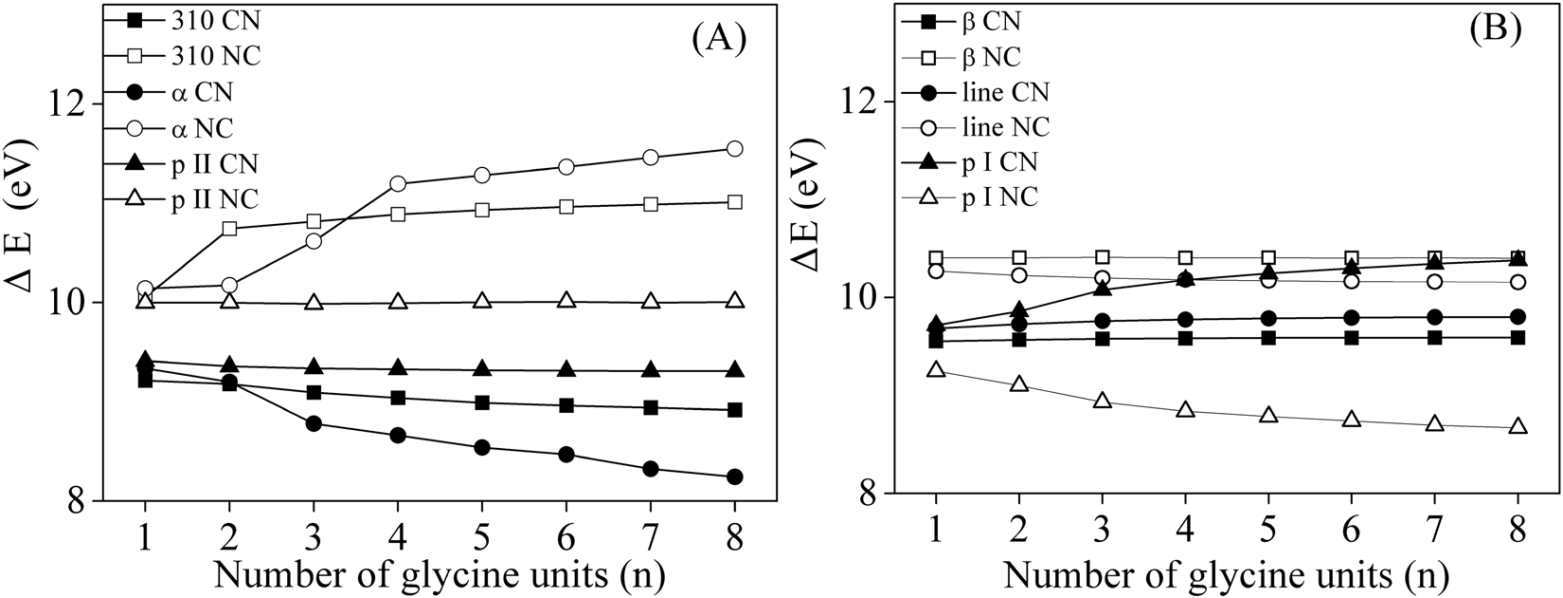
Bidirectional HOMO-LUMO gaps of (**A**) 3_10_-helix, α-helix, polyproline helical II and (**B**) β-sheets, linear, polyproline helical I structures.

**Figure 5:**
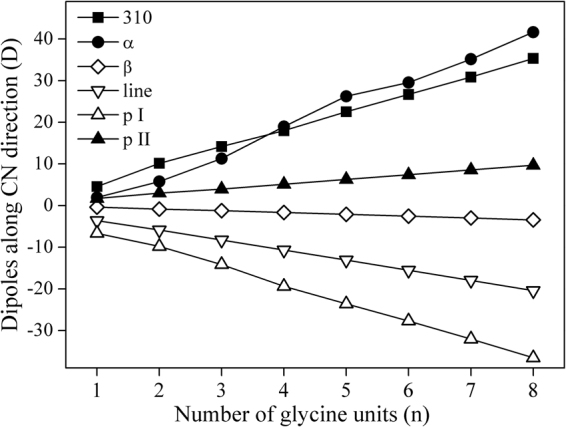
Molecular dipoles along the CN direction plotted against the number of glycine units (n).

In this work, the neutral methylene radicals were used as donors and accepters. It allows us to analyze the bidirectional gaps (ΔE) between the lowest unoccupied molecular orbital (LUMO) and the highest occupied molecular orbital (HOMO) of the same polypeptide chain, and the differences should be only attributed to the structural transitions. In Fig. 4, the bidirectional HOMO-LUMO gaps of different secondary structures were plotted with the number of glycine units (n). For the 3_10_-helix, α-helix and polyproline II helical structures (defined as type A hereafter), the ΔE values in CN direction decrease with the increase of glycine units, while HOMO-LUMO gaps at NC direction increase. The amplitude of ΔE changes is in the same order of α-helix> 3_10_-helix> polypro II. For β-strand, linear and polyproline I helical structures (defined as type B), the ΔE values in CN (NC) direction increase (decrease) with the increasing glycine units. And the amplitudes decrease in the order of polypro I> linear> β-strand. The changing trend can be correlated well with the molecular dipole moments. In Fig. 5, the molecular dipoles in CN direction were shown against the glycine number for various secondary structures. In type A structures, the positive dipole moments increase with the increasing chain lengths in the order of α-helix> 3_10_-helix> polypro II. In type B structures, the dipole moments in CN direction are negative and decrease with the increase of glycine units in the order of polypro I> linear> β-strand. In a word, the increasing positive dipole moments cause the decreasing ΔE in CN direction, while the decreasing negative dipoles lead to increasing ΔE values.

As to the bidirectional ET, the HOMO-LUMO gaps in CN direction are obviously lower than that in opposite direction, except for polypro I. According to Barton’s hypothesized model, ET occurs in direction CN (NC) when MutY binds to (dissociates from) DNA duplex. In the MutY core fragment (PDB code: 1KQJ)^58^, 62.5% of the total residues form 15 α-helices. As a result of much lower ΔE in CN direction, the binding between protein and DNA would take place easier, and the dissociation process should be activated by another redox enzyme. It confirms the rationality of the repair mechanism proposed by Barton *et al*.

## Conclusions

In the present work, the MTBC model was further refined to reflect the influences of structural transitions on ET rate more quantitatively. With this model, various polyglycine structures, which are terminated by neutral methylene radicals, were selected to investigate the protein-mediated bidirectional ET. According to the electronic structure analyses, the secondary structures can be divided into two types; i.e., A with positive dipoles in CN direction (α-helix, 3_10_-helix and polypro II) and B with negative dipoles in the same direction (β-strand, linear and polypro I). For type A, the HOMO-LUMO gaps (ΔE) in CN direction decrease with the increasing glycine units, and the decreasing ranges decrease in the same order of positive dipoles (α-helix> 3_10_-helix> polypro II). As to type B, similar trend occurs but in the opposite direction NC. As to the bidirectional ET, the HOMO-LUMO gaps in CN direction are obviously lower than that in opposite direction, except for polypro I. Thus, the ET would take place easier in CN direction. However, as to the ET with the same tunneling energy, the differences between bidirectional coupling strengths are slight for all structures. It provides the theoretical support for the shuttle function of protein molecular wire and the rapid dissociation and redistribution model of MutY in efficient DNA damage detection.

Furthermore, with the refined MTBC model, the influences of structural transitions on ET rate were also investigated. It was found that the coupling strengths are not only affected by the Ramachandran angles, but also correlated to the alignment of C=O vectors, the alignment of peptide planes and the rearrangement of other structure factors. The results here would provide a theoretical evidence for the coupled dynamics of proteins, and can be used to rationalize the differences of ET across different protein structures and design more efficient protein molecular wires.
